# The accessory adapters FAF1, FAF2, and UBXN7 accelerate proteasomal degradation by increasing prior p97-mediated substrate unfolding

**DOI:** 10.1126/sciadv.aea7381

**Published:** 2026-03-06

**Authors:** Matthias Kracht, Alexander Kröning, Johannes van den Boom, Pinki Gahlot, Sandra Koska, Leo Kiss, Hemmo Meyer

**Affiliations:** ^1^ Molecular Biology I, Center of Medical Biotechnology, Faculty of Biology, University of Duisburg-Essen, 45141 Essen, Germany.; ^2^ Department of Molecular Machines and Signaling, Max Planck Institute of Biochemistry, Martinsried 82152, Germany.

## Abstract

The AAA-ATPase VCP/p97 with its adapter Ufd1-Npl4 unfolds ubiquitylated substrate proteins to prepare degradation in the proteasome; however, the function of critical accessory factors remains unclear. Here, we show in the mammalian system that efficient protein degradation in the proteasome requires accessory adapters that boost p97-mediated unfolding likely by positioning Ufd1 for substrate loading. In a reaction that reconstitutes p97-Ufd1-Npl4–mediated unfolding coupled to proteasomal degradation, degradation was inefficient but stimulated by accessory adapters FAF1, FAF2, or UBXN7. Stimulation of proteasomal degradation was largely caused by an increase of p97 unfolding rates, conveyed by a helix-UBX segment in FAF1/2 that tethered the UT3 ubiquitin binding module of Ufd1 to the p97 N-domain. Mutations that abrogated the helix-Ufd1 interaction reduced stimulation of degradation, suggesting that accessory adapters position Ufd1 within the p97 complex to organize proficient substrate loading. Our results define the function of accessory adapters in mammals and highlight the complexity of substrate loading onto p97 for efficient substrate processing.

## Teaser

A group of accessory adapters positions the p97 adapter Ufd1 to boost substrate unfolding and subsequent proteasomal degradation.

## INTRODUCTION

The hexameric AAA adenosine triphosphatase (ATPase) p97 (also called valosin-containing protein (VCP), or Cdc48 in yeast) is an essential component of the ubiquitin-proteasome system as it binds and unfolds a subset of ubiquitylated proteins to prepare them for degradation in the proteasome (*1*–*3*). Each p97 subunit has two AAA domains (D1 and D2) that form stacked rings with a regulatory N-terminal domain (N-domain) positioned at the D1 ring. Substrate proteins are unfolded by threading them through the central pore of the p97 hexamer in a process driven by adenosine 5′-triphosphate (ATP) hydrolysis (*4*–*8*). For substrate recruitment and loading into the pore, p97 requires a primary heterodimeric adapter formed by ubiquitin fusion degradation protein 1 (Ufd1) andnuclear protein localization protein 4 homolog (Npl4) (*4*, *9*–*11*). Ufd1-Npl4 binds the ubiquitin chain conjugated to the substrate, identifies an initiator ubiquitin, and inserts the initiator ubiquitin into the central pore for further threading by p97 (*4*, *12*). p97 thus unfolds and prepares substrate proteins for degradation in the proteasome. This preparation is particularly critical for substrates that lack disordered peptide stretches required for processing by the proteasome (*13*, *14*).

Besides Ufd1-Npl4, however, a host of additional p97 cofactor proteins exist that bind and assist p97. Among them, a group of proteins, comprising FAS-associated factor 1 (FAF1), FAS-associated factor 2 (FAF2), and UBX domain-containing protein 7 (UBXN7), shares an overall similar domain architecture with an N-terminal ubiquitin-binding ubiquitin-associated (UBA) domain, a central ubiquitin-associated sequence (UAS) thioredoxin-fold domain of unknown function and a C-terminal ubiquitin-regulatory X (UBX) domain as a binding module for the p97 regulatory N-domain (*15*, *16*). The three factors assist the p97-Ufd1-Npl4 complex in unfolding ubiquitylated substrate proteins in important cellular signaling and protein quality control pathways. FAF2 (also called UBXD8 or Ubx2) is anchored to endomembranes through a hairpin loop and helps p97 extract membrane proteins in pathways such as endoplasmic reticulum–associated degradation (*17*–*19*). In contrast, UBXN7 and FAF1 have important chromatin-associated roles during DNA replication and repair such as assisting p97-Ufd1-Npl4 in extracting the replicative helicase minichromosome maintenance complex component 2-7 (MCM2-7) at termination of replication (*20*–*24*). Despite the relevance of FAF1, FAF2, and UBXN7 as accessory adapters in fundamental cellular processes, their molecular function during p97-Ufd1-Npl4–mediated unfolding of ubiquitylated substrate proteins and its importance for efficient downstream proteasomal degradation has remained controversial (*25*, *26*).

In this study, we used purified mammalian components to reconstitute p97-mediated unfolding of a ubiquitylated substrate coupled to downstream proteasomal degradation. We find that the accessory adapters stimulate proteasomal degradation by boosting p97-mediated unfolding rates and that this is achieved by binding Ufd1 likely to control the position of Ufd1 within the p97-Ufd1-Npl4 complex.

## RESULTS

### Reconstitution of a coupled unfolding-degradation reaction

**Fig. 1.**
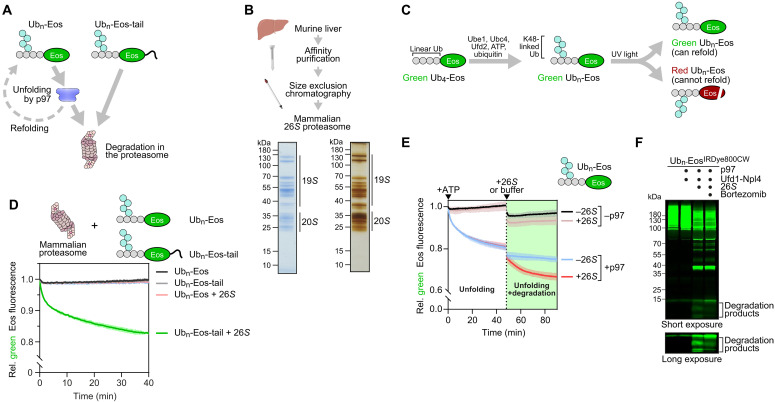
A coupled unfolding-degradation reaction with mammalian VCP/p97 and the proteasome. (**A**) Proteasomal degradation of ubiquitylated proteins with a disordered tail occurs directly, whereas degradation of tightly folded proteins requires preprocessing by p97. Substrates can refold after unfolding if not degraded by the proteasome. (**B**) The proteasome used in this study was affinity purified from murine liver extract followed by size exclusion chromatography. The peak fraction is shown in Coomassie- and silver-stained SDS gels. (**C**) A model substrate harboring four ubiquitin moieties fused to green-fluorescent Eos was purified and enzymatically poly-ubiquitylated with K48-linked ubiquitin chains. A backbone break induced by ultraviolet (UV) irradiation in a fraction of the sample leads to a shift to red fluorescence and prevents substrate refolding. (**D**) Time course of fluorescence intensities of poly-ubiquitylated Ub_4_-Eos with or without a short disorder C-terminal peptide tail incubated with the proteasome. Note that only the substrate with tail is degraded by the proteasome indicated by the loss of green fluorescence. *n* = 3. (**E**) A coupled unfolding-degradation reaction with components added as indicated. Note that, during an initial incubation with p97 and Ufd1-Npl4, fluorescence declines and reaches a plateau indicating unfolding and refolding. Addition of the proteasome further reduces fluorescence because of substrate degradation. *n* = 3. (**F**) SDS-gel analysis of fluorescently labeled substrate incubated with the indicated components. Note the generation of proteolytic peptides in the presence of p97-Ufd1-Npl4 and the proteasome that is sensitive to the proteasome inhibitor bortezomib.

The 26*S* proteasome can process ubiquitylated substrate proteins with a disordered peptide stretch directly, while substrates lacking such a stretch require preprocessing by p97 (Fig. 1A). In an effort to understand how mammalian p97 and 26*S* proteasome cooperate, we reconstituted a coupled protein unfolding-degradation reaction from purified components. We affinity purified the 26*S* proteasome from mouse liver using a glutathione *S*-transferase (GST)–fusion with the ubiquitin-like (UBL) domain of RAD23B and eluted with the ubiquitin-interacting motif (UIM) domain derived from 26*S* proteasome non-ATPase regulatory subunit 4 (PSMD4) followed by size exclusion chromatography (Fig. 1B) (*27*). The purified proteasome was active in degrading a minimal model substrate comprising fluorescent Eos with an in-line N-terminal fusion of four ubiquitin moieties (fig. S1, A and B). Because p97 requires longer ubiquitin chains, we enzymatically poly-ubiquitylated the reporter for all further experiments (Fig. 1C). As expected, the proteasome alone degraded the ubiquitylated substrate containing a disordered tail, but not a tailless substrate known to require prior unfolding by p97 (Fig. 1D). We therefore used the poly-ubiquitylated reporter without the tail to monitor the dependency of degradation on p97 in all subsequent assays. We conducted the coupled reaction in two stages. In the first stage, incubation with p97 and Ufd1-Npl4 led to an initial loss of fluorescence because of substrate unfolding but then reached a plateau representing a steady state between unfolding and refolding (Fig. 1E), as previously reported (*28*, *29*). Of note, upon subsequent addition of proteasome to the reaction, substrate fluorescence further declined below the steady-state level (Fig. 1E), indicating that the 26*S* proteasome degraded the substrate that was unfolded by the p97-Ufd1-Npl4 complex. In contrast, in reactions lacking p97, the proteasome did not notably affect fluorescence of the tailless substrate, as expected. Substrate degradation in the presence of p97-Ufd1-Npl4 and the proteasome was reflected by the generation of peptides from the fluorescently labeled substrate as detected in SDS gels, and this was sensitive to addition of a proteasome inhibitor, as expected (Fig. 1F). While this demonstrated that the coupled reaction was proficient, we noted that unfolding and subsequent degradation of the reporter were relatively inefficient compared to activities of the yeast Cdc48/p97 reported earlier (*11*, *29*).

### A group of accessory p97 cofactor proteins stimulates degradation by boosting p97-mediated unfolding

**Fig. 2.**
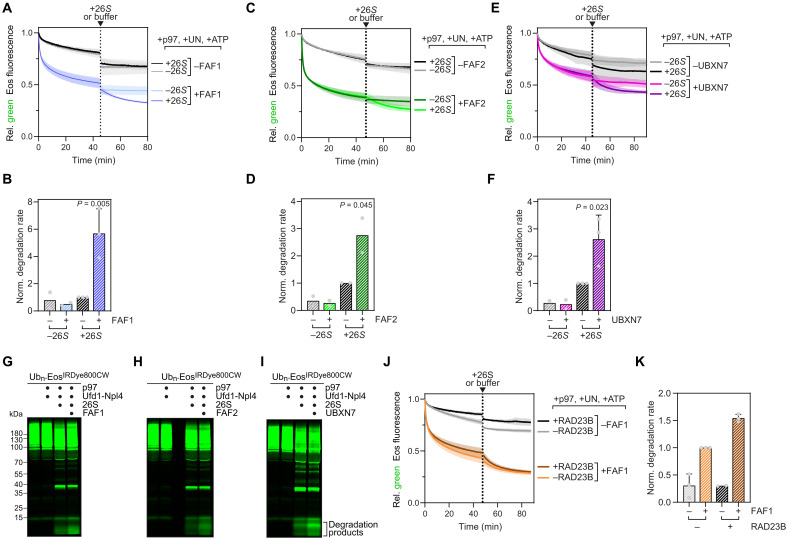
Accessory adapters FAF1, FAF2, and UBXN7 stimulate p97-dependent degradation in the proteasome by increasing p97-mediated unfolding. (**A**) The substrate was first incubated with p97 and Ufd1-Npl4 in the presence or absence of FAF1. After 45 min, the proteasome or buffer alone was added. Note the stimulation of substrate unfolding by FAF1 in the first stage, and the increased degradation upon proteasome addition. *n* = 2. UN, Ufd1-Npl4. (**C** and **E**) Equivalent experiments as in (A) with FAF2 and UBXN7, respectively. *n* = 2. (**B**, **D**, and **F**) Quantifications of initial degradation rates in (A), (C), and (E). *n* ≥ 2. *P* values indicate significance of difference from control (+26*S* without accessory adapter) using one-way analysis of variance (ANOVA). Error bars represent SD. (**G**, **H**, and **I**) SDS-gel–based analysis of the generation of proteolytic peptides from fluorescently labeled substrate by the proteasome with p97, Ufd1-Npl4, and the indicated accessory adapters. (**J**) A coupled unfolding-degradation reaction in the presence of FAF1 and the proteasomal shuttling factor RAD23B as indicated. Note that RAD23 stimulated degradation also in the presence of FAF1. *n* = 3. (**K**) Quantification of initial degradation rates in (J). *n* = 3. Error bars represent SD.

To explain the low efficiency, we turned to a group of related accessory adapters, comprising FAF1, FAF2, and UBXN7 that assist p97-Ufd1-Npl4 in various cellular pathways in higher eukaryotes. We asked whether the accessory adapters may cooperate to account for full p97 unfolding activity and proteasomal degradation in the mammalian system. When added to our coupled assay, any of the three cofactors significantly stimulated proteasomal degradation rates in the second stage of the reaction by 2.5- to 5-fold compared to reactions without the accessory adapters (Fig. 2, A to F). This again was reflected by the generation of proteolytic peptides, which was stimulated by FAF1, FAF2, or UBXN7 in reactions containing p97-Ufd1-Npl4 and proteasome (Fig. 2, G to I). Of note, the accessory factors also largely accelerated the loss of reporter fluorescence already in the first stage of the reaction before addition of the proteasome, indicating an increase of the unfolding rate (Fig. 2, A, C, and E). This stimulation did not occur with accessory adapters alone as unfolding was dependent on the presence of Ufd1-Npl4, as expected (fig. S2, A and B). The stimulation by an accessory adapter also occurred at higher concentrations of p97, cofactors, and substrate closer to the physiological concentrations (fig. S2, C and D). As a result of unfolding stimulation, the steady state between unfolding and refolding in the plateau phase shifted in favor of the unfolded state (Fig. 2, A, C, and E). This indicates that the accessory cofactors cause increased degradation rates by accelerating substrate unfolding.

Using an end-point assay that scored the mobilization of the MCM2-7 helicase from DNA, it was previously observed that FAF1, FAF2, and UBXN7 help p97-Ufd1-Npl4 process substrates with shorter ubiquitin chains (*25*). By analyzing unfolding rates instead, we confirmed lower unfolding rates for substrates with shorter ubiquitin chains, as expected (fig. S2, E to I), but noted that the relative stimulation of the processing of substrate with different chains lengths was comparable for all chain lengths, rendering it unlikely that the accessory adapters increase substrate affinity only and specifically for shorter chains.

Intriguingly, the ortholog of UBXN7 in yeast, Ubx5, was reported to regulate yeast Cdc48-dependent degradation in the proteasome, but an increase in unfolding rates could not be observed (*26*). We confirmed that Ubx5 did not stimulate substrate unfolding by yeast Cdc48 and yeast Ufd1-Npl4 (fig. S3, A and B), nor did any of the human accessory adapters stimulate Cdc48 with yeast Ufd1-Npl4 (fig. S3C), indicating a difference between yeast and mammalian systems. Consistent with that, yeast Cdc48 cooperated with human Ufd1-Npl4 but was much less active when compared with the yeast Ufd1-Npl4 counterpart (fig. S3, D and E). Of note, unfolding by yeast Cdc48 with human Ufd1-Npl4 was largely stimulated by the human accessory adapters, in the case of FAF2 even reaching almost full activity of yeast Cdc48-Ufd1-Npl4 (fig. S3, D and E). This indicates that it is the mammalian Ufd1-Npl4, and not p97 itself, that acquired a dependency on stimulation by the accessory adapters when compared to yeast Ufd1-Npl4.

Li and colleagues proposed that, in yeast, the accessory adapter Ubx5 increases the affinity of Cdc48 to the substrate and, thereby, helps overcome nonproficient direct targeting of Cdc48-dependent substrates to the proteasome by proteasomal shuttling factors (*26*). However, in the mammalian system, we did not observe a major increase in binding of the ubiquitylated reporter in the presence of FAF1 in pulldowns of p97-Ufd1-Npl4 (fig. S3F). Using a more sensitive fluorescence polarization approach, we measured a twofold FAF1-induced increase in the affinity of the p97 complex to fluorescently labeled free lysine-48–linked ubiquitin chains (fig. S3, G and H). Consistent with that, using biolayer interferometry, we detected a roughly 2.5-fold increased on-rate in binding of p97-Ufd1-Npl4 to our ubiquitylated substrate when FAF1 was present, suggesting that initial binding of the substrate to p97 is improved by FAF1 (fig. S3I). Moreover, although addition of the human proteasomal shuttling factor RAD23B slightly reduced the rate of p97-driven unfolding in the absence or presence of FAF1 before addition of the proteasome, this reduction was minor compared with the stimulation of unfolding by FAF1 (Fig. 2, J and K). Degradation was stimulated when both factors, FAF1 and RAD23B, were present (Fig. 2, J and K). These results suggest that mammalian accessory adapters increase proteasomal degradation of p97-dependent substrates mainly by stimulating p97-mediated unfolding rates.

### A minimal helix-UBX fragment of FAF1 and FAF2 conveys stimulation of unfolding

**Fig. 3.**
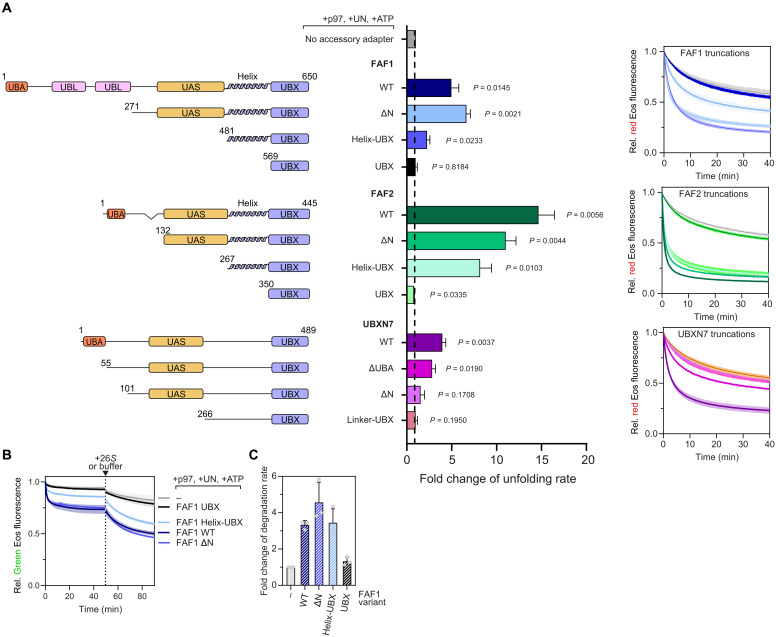
Structure-function analysis of the stimulatory effect on unfolding by the accessory adapters FAF1, FAF2, and UBXN7. (**A**) Indicated truncations of accessory adapters on the left were assayed for their effect on p97 and Ufd1-Npl4–mediated unfolding. Reaction curves are shown on the right. Corresponding quantifications are depicted in the middle. Note that the helix-UBX fragments lacking UBA and UAS domains of FAF1 and particularly FAF2 retain stimulatory activity. *P* values indicate significance of difference from control without accessory adapter using one-sample *t* test with a hypothetical value of 1. *n* = 3. UBA, ubiquitin-associated; UAS, ubiquitin-associated sequence; UBX, ubiquitin-regulatory X. (**B**) Effect of indicated FAF1 fragments on coupled unfolding-degradation reactions, revealing stimulatory activity of the helix-UBX fragment also on degradation. *n* = 3. UN, Ufd1-Npl4. (**C**) Quantification of (B). *n* = 3.

To understand the mechanism underlying the stimulatory effect, we aimed to identify the minimal structural elements in the accessory adapters that mediate the increase of unfolding rates by the p97-Ufd1-Npl4 complex. All three accessory adapters comprise an N-terminal ubiquitin-binding UBA domain, a thioredoxin-fold UAS domain in the middle, and a helical region followed by the p97-binding UBX domain at the C terminus. Sequential truncations revealed that the activity of FAF1 did not rely on its UBA domain or other elements N-terminal of the UAS domain, including two UBL domains in FAF1, and an N-terminal deletion only had a minor effect on FAF2 (Fig. 3A). Even deletion of the UAS domain, leaving only the UBX domain with the helical extension, had little effect on FAF2 and still retained a significant stimulatory effect in the case of FAF1 (Fig. 3A). In contrast, the UBX domains alone without the helical extension had no activity (Fig. 3A). Despite its overall similar domain structure, UBXN7 behaved differently (Fig. 3A). Loss of the UBA domain had a gradual effect already, while further deletions completely abolished UBXN7’s stimulatory activity, suggesting that UBXN7 requires more sequence elements than FAF1 or FAF2 to act on the p97-Ufd1-Npl4 complex. Consistent with the increase in the unfolding rate underlying accelerated proteasomal degradation, the helix-UBX fragment of FAF1 alone conveyed full stimulatory activity in the coupled unfolding-degradation reaction (Fig. 3, B and C).

### The helix-UBX elements of FAF1 and FAF2 form a strut that links the p97 N-domain with the Ufd1 globular domain

**Fig. 4.**
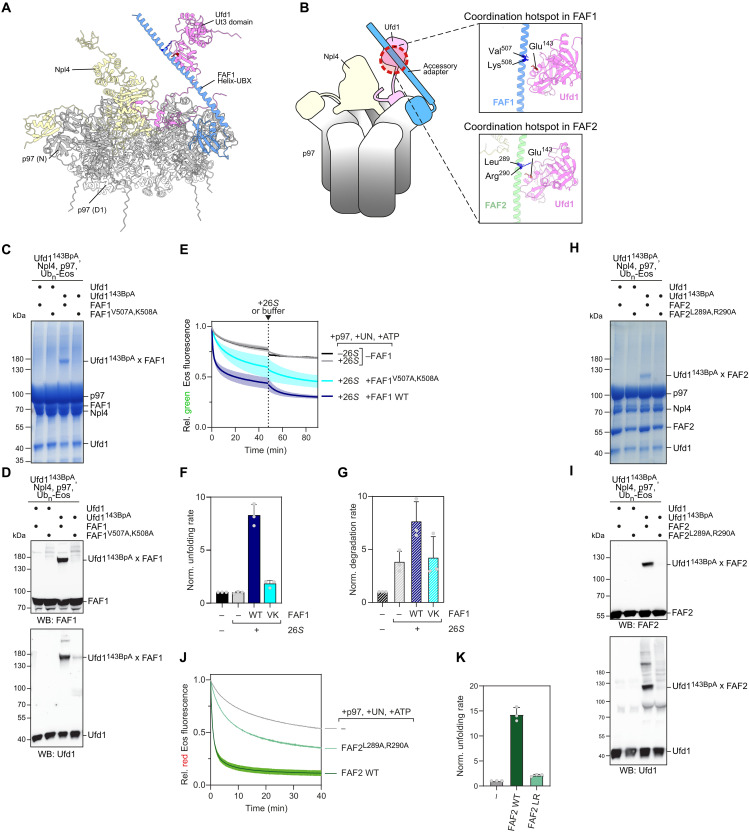
Interaction of the Ufd1 UT3 domain with the helical struts of FAF1 and FAF2 is critical for the stimulation. (**A**) AlphaFold 3 model of p97 (ND1), Ufd1-Npl4, and the FAF1 helix-UBX fragment. Note the contact site of the Ufd1 UT3 domain with the FAF1 helix in addition to the expected binding of the UBX domain to the p97 (N) domain. (**B**) Cartoon depiction of the predicted structures with blow-up of the contact sites of Ufd1 with FAF1 or FAF2 indicating critical residues, respectively. (**C**) Confirmation of FAF1-Ufd1 contact site by site-specific cross-linking. Ufd1 wild type or Ufd1 harboring the photocrosslink amino acid BpA at position 143 was incubated with FAF1 wild type or the FAF1 V507A, K508A mutant predicted to interfere with Ufd1 binding. The cross-link was induced by UV irradiation, and samples were analyzed in a Coomassie-stained SDS gel. Note that the V507A, K508A mutation abrogates FAF1-Ufd1 interaction. (**D**) Western blot (WB) analysis of cross-linked samples in (C) with antibodies specific for FAF1 or Ufd1 as indicated. (**E**) Coupled unfolding-degradation reactions comparing FAF1 and FAF1 V507A, K508A as indicated. Note that the V507A, K508A mutation reduced stimulation by FAF1. *n* = 3. (**F** and **G**) Coupled unfolding-degradation rates with FAF1 wild type and mutant as indicated as measured in (E). Quantification of initial unfolding and degradation rates, respectively. *n* = 3. Error bars represent SD. (**H**) Confirmation of FAF2-Ufd1 contact site by site-specific cross-links as shown for FAF1 in (C) with FAF2 wild type and the FAF2 L289A, R290A mutant. (**I**) Western blot analysis of cross-linked samples in (H) with antibodies specific for FAF2 or Ufd1 as indicated. (**J** and **K**) Unfolding reactions comparing FAF2 and FAF2 L289A, R290A as indicated. Note that the L289A, R290A mutation reduced stimulation by FAF2. Quantification of initial unfolding rates. *n* = 3.

For further insight, we turned to AlphaFold 3 to predict the structure of p97 (ND1) with Ufd1-Npl4 and the active helix-UBX fragments of FAF1 or FAF2 (Fig. 4, A and B; and fig. S4, A to D). Of note, in available cryo–electron microscopy structures, Npl4 is positioned on the p97 hexamer above the central entry pore, but the globular UT3 domain of Ufd1 is not visible. In our prediction, the UBX domain of both FAF1 and FAF2 binds to one of the p97 N-domains, while the helical domain, which is evolutionarily conserved (fig. S4E), extends up like a rigid strut to the center diagonally above Npl4 where it binds and possibly positions the Ufd1 UT3 domain (Fig. 4, A and B). In support of this notion, comparison of several AlphaFold 3 models showed that the Ufd1 UT3 domain was, on average, positioned closer to Npl4 and the positions deviated less in the presence than in the absence of FAF1 (fig. S4, F and G). To confirm this prediction experimentally, we introduced the genetically encoded photocrosslinker BpA at residue 143 of Ufd1 at the interface of the UT3 domain with the FAF1 helix. Ufd1-143BpA efficiently cross-linked to FAF1 as confirmed by Western blot and as detectable even in Coomassie-stained gels (Fig. 4, C and D). Of note, the cross-link was abolished by mutation of critical residues in the predicted opposing FAF1 interface, V507 and K508, to alanines, thus confirming the interaction with Ufd1 (Fig. 4, C and D). Consistent with this, FAF1 harboring the V507A, K508A mutations had a largely reduced stimulatory activity in substrate unfolding and, consequently, in subsequent substrate degradation by the proteasome (Fig. 4, E to G). Moreover, in cells, overexpression of the mutant FAF1 (V507A, K508A) protein induced an increase in cell death compared to overexpression of wild type FAF1 (fig. S4H), confirming physiological relevance of the FAF1-Ufd1 interface in FAF1’s functions in cell cycle regulation. Equivalent biochemical experiments were carried out for FAF2 with the L289A and R290A mutations, abolishing Ufd1-BpA143 cross-links and reducing the stimulating effect on p97-mediated unfolding (Fig. 4, H to K). Thus, FAF1 and FAF2 apply a similar structural mechanism to position Ufd1 within the p97-Ufd1-Npl4 complex and thus stimulate unfolding of ubiquitylated substrates.

## DISCUSSION

**Fig. 5.**
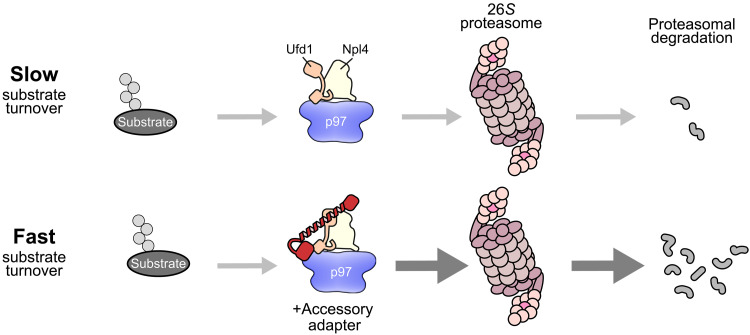
Cartoon model. In the absence of an accessory adapter, unfolding and degradation rates are low. In the presence of an accessory adapter, stabilization of Ufd1 by the p97 N-domain–anchored helix increases unfolding and, thus, degradation.

The abundant unfoldase VCP/p97 is essential for a plethora of pathways that rely on the efficient degradation of proteasomal substrate proteins particularly in stress conditions. Ufd1-Npl4 is the primary p97 adapter for ubiquitylated substrates and helps funnel them into the central pore of p97 for substrate threading and concomitant unfolding (*4*, *30*). In this study, we have clarified the molecular role of a class of critical accessory adapters comprising FAF1, FAF2, and UBXN7 and unraveled how they increase the efficiency of proteasomal degradation. On the basis of direct biochemical evidence in an assay that couples unfolding with degradation in the mammalian system and follows reaction rates rather than end points, we show that the accessory adapters boost proteasomal degradation by increasing the rate of p97-mediated substrate unfolding and that this, at least for FAF1 and FAF2, is mediated by a structural element that links the globular UT3 domain of Ufd1 to one of the p97 N-domains (Fig. 5).

In the yeast system, it has been shown that accessory adapters such as Ubx5 improve proteasomal degradation of Cdc48/p97-dependent substrates by prioritizing substrate targeting first to Cdc48/p97 and thus possibly preventing futile shuttling to the proteasome before processing by Cdc48/p97 (*26*). Of note, we find that the mammalian accessory adapters, unlike Ubx5 in the yeast system, stimulate the rate of substrate unfolding at the level of p97 already and that this occurs even in the absence of the proteasome. Moreover, the presence of the proteasomal shuttling factor RAD23B only mildly slowed down substrate unfolding by p97 arguing that competition between p97 and proteasomal shuttling is not as crucial in the mammalian system. This suggests that it is largely the acceleration of p97-mediated substrate unfolding by accessory adapters that helps boost proteasomal degradation of p97-dependent substrates.

Increased unfolding rates by the accessory adapter could be mediated through improved ubiquitin binding and substrate recruitment, although we observed only an about twofold increase in affinity and on-rates of the p97 complex by FAF1, and the Ufd1-Npl4 adapter already has ubiquitin binding regions (*4*, *9*, *31*). Notably, our structural predictions, confirmed by cross-linking experiments, show that the UBX domain of FAF1 and FAF2 locks onto a p97 N-domain and that a helix extends as a strut from that UBX domain to the center diagonally above p97 and Npl4 where the helix binds Ufd1. It is possible that UBXN7 mediates a similar effect with an alternative structural mechanism. The interaction of accessory adapters with Ufd1 is intriguing. Ufd1 is anchored via its disordered C-terminal region both to a p97–N-domain and to Npl4 (*4*, *32*, *33*). However, the N-terminal globular UT3 domain of Ufd1 is not resolved in structures of the p97-Ufd1-Npl4 complex, suggesting that it is flexible in the absence of accessory adapters even in the presence of the substrate.

Of note, the UT3 domain binds ubiquitin in a region that does not overlap with the FAF1/2 binding site (*31*) and helps choose the suitable ubiquitin moiety within the ubiquitin chain on the substrate to initiate unfolding (*12*). Correct positioning of the UT3 domain by FAF1/2 may, therefore, assist with identification of the initiator ubiquitin and may help position the remaining ubiquitin chain and substrate for subsequent efficient processing by p97. In this scenario, the role of the accessory adapters would be to bring UT3 and, thereby, the ubiquitin chain in the correct position, possibly even by transferring forces that are generated by up-and-down movements of the p97 N-domain. This would ensure efficient substrate threading after initial insertion into the p97 pore and result in the observed increased rates of unfolding and subsequent degradation as required in many cellular pathways. A recent paper, published during revision of this manuscript (*34*), reported direct binding of ubiquitin to the FAF1/2 helix in a region that overlaps with the UT3 binding site in FAF1/2 reported here. It is, therefore, conceivable that the ubiquitin chain of the substrate may also bind to FAF1/2 directly possibly before the UT3 locks onto the helix and, by interaction with the ubiquitin chain, positions the substrate in a favorable manner for processing by p97.

## MATERIALS AND METHODS

### Purification of mouse 26*S*-proteasome

Murine 26*S* proteasome was purified from liver using the UBL-affinity method (*27*). Livers were taken from unused wildtype C57BL/6J mice (2 to 6 months old) according to the institutional guidelines of the University Duisburg-Essen and approved by the University’s animal welfare committee. Mouse husbandry was approved by the city of Essen (Az: 32-2-11-80-71/203). The entire procedure was performed at 4°C. Livers were cut into small pieces and homogenized in 6 ml of UBL buffer [25 mM Hepes (pH 7.4), 5 mM MgCl_2_, 10% glycerol, 2 mM ATP, and 1 mM dithiothreitol (DTT)] per gram of liver using a potter homogenizer. The lysate was cleared by a short spin (10 min) at 1500*g* followed by a long spin (1 hour) at 100,000*g*. The supernatant was supplemented with 0.05 ml of glutathione-Sepharose (Cytiva) per milliliter of lysate and mixed for 15 min to deplete endogenous GST. The mixture was loaded on a gravity flow column with a filter, and the flow through was supplemented with fresh glutathione-Sepharose (0.05 ml per milliliter of lysate) and GST-UBL from RAD23B (1-82) (0.2 mg/ml). The solution was incubated for 1 hour under constant overhead turning and subsequently filtered through a fresh gravity filter column. The retained glutathione-Sepharose was washed two times with 5 ml of UBL buffer, and bound 26*S* proteasome was eluted with 2× bed volume of UBL buffer containing His-UIM-Strep (2 mg/ml). The eluate was further purified using a Superose 6 size exclusion column (Cytiva). High–molecular weight fractions were analyzed by silver stain and tested for proteolytic activity using Suc-LLVY-AMC (AAT Bioquest). Fractions containing 26*S* proteasome were pooled, concentrated, supplemented to 40°C glycerol, and stored at −20°C.

### Expression of recombinant proteins

All expression constructs are listed in table S1. The accessory factor sequences (FAF1, FAF2, UBXN7, and Ubx5) were cloned into the pET28 vector bearing an N-terminal poly-His tag. For FAF2 and its mutants, the transmembrane domain amino acids 90 to 118 were deleted in every variant to prevent solubility issues. RAD23B was cloned into pGEX6P1, bearing a GST tag and a precision protease cleavage site. All proteins except His-p97 and Ufd1-His E143BpA were expressed in *Escherichia coli* Rosetta 2 (DE3). Cells were grown at 37°C to an optical density at 600 nm (OD_600_) of 0.6 to 0.8, induced with 0.5 mM isopropyl-β-d-thiogalactopyranoside (IPTG), and grown further at 18°C overnight. After harvesting by centrifugation (4000 rpm), cell pellets were resuspended in buffer A [50 mM Hepes (pH 8.0), 150 mM KCl, 2 mM MgCl_2_, and 5% glycerol] and stored at −80°C. For His-p97, High Five insect cells were infected with at baculovirus coding for His-p97 (human) and grown for 4 days at 27°C. Cell pellets were harvested by centrifugation (1000 rpm), washed once with ice cold 1× phosphate-buffered saline (PBS), and resuspended in buffer A before storage at −80°C. For Ufd1-His E143Bpa, *E. coli* Bl21 (DE3) were cotransfected with a plasmid with amber stop codon (nucleotide sequence TAG) instead of the codon for glutamate at position 143 in Ufd1-His and the pEVOL-pBpf plasmid (Addgene, plasmid no. 31190) (*35*). Cells were grown at 37°C to an OD_600_ of 0.6 to 0.8; induced with 0.5 mM IPTG, 0.2% arabinose, and 1 mM *p*-benzoyl-l-phenylalanine; and grown further at 18°C overnight. After harvesting by centrifugation (4000 rpm), cell pellets were resuspended in buffer A and stored at −80°C.

### Purification of His-p97, p97-His, and His-Cdc48

Cell pellets were thawed and supplemented with phenylmethylsulfonyl fluoride (1 mM), 20 mM imidazole, and lysozyme (1 mg/ml) and incubated at 4°C for 30 min, followed by lysis through sonication (5× 30-s pulses with 30 s of pause between each pulse). Lysates were centrifuged for 1 hour at 4°C with 30,000*g*, and the supernatant was filtered. The cleared lysate was then passed through a HisTrap column (Cytiva). The column was washed with 200 ml of buffer A and 20 mM imidazole, and bound protein was eluted with 25 ml of buffer A containing 300 mM imidazole. The eluate was further purified by ion-exchange chromatography, using a HiTrap Q HP column (Cytiva) with a salt gradient from 150 mM to 1 M KCl in Buffer A. Fractions containing p97 were pooled, concentrated with a 100-kDa molecular weight cut-off (MWCO) spin column (Sartorius), diluted 1:10 with Buffer B [50 mM Hepes (pH 7.4), 150 mM KCl, 2 mM MgCl_2_, and 5% glycerol], and concentrated again. This was repeated twice to remove excess salt. Unless noted otherwise, His-p97 expressed in insect cells was used in all experiments.

### Purification of His-tagged cofactors and substrates

For Ufd1-His and Npl4, lysates of Ufd1-His and Npl4 were combined before purification (*36*). Lysates were centrifuged for 1 hour at 4°C with 30,000*g*, and the supernatant was filtered. The cleared lysate was then passed through a HisTrap column (Cytiva). The column was washed with 200 ml of buffer A and 20 mM imidazole, and bound protein was eluted with 25 ml of the same buffer containing 300 mM imidazole. The eluate was further purified by size exclusion chromatography using a S200 16/600 gel filtration column (Cytiva) with a running buffer containing buffer B and 1 mM DTT.

### Purification of RAD23B

The lysate was centrifuged for 1 hour at 4°C with 30,000*g*, and the supernatant was filtered. The cleared lysate was then passed through a GSTrap column (Cytiva). The column was washed with 50 ml of buffer A, and bound protein was eluted with 25 ml of buffer A and 20 mM glutathione. The eluate was supplemented with 1 μg of GST-PreScission protease per 6 μg of protein and dialyzed in buffer B and 1 mM DTT overnight. Cleaved GST tags and GST-PreScission protease were removed by passage through a GSTrap column (Cytiva), and the flow through was further purified by size exclusion chromatography using a S200 16/600 gel filtration column (Cytiva) with the dialysis buffer.

### Purification of ubiquitin

The lysate was centrifuged for 1 hour at 4°C with 30,000*g*, and the supernatant was supplemented with 0.5% perchloric acid, incubated for 10 min, and centrifuged again. This second supernatant was dialyzed with 2 liters of 25 mM ammonium acetate (pH 4.5) overnight, before passage through a SP HP cation exchange column (Cytiva). The column was washed with 50 ml of 25 mM ammonium acetate (pH 4.5) and eluted with a gradient from 25 mM ammonium acetate (pH 4.5) to 250 mM ammonium acetate (pH 7.6) and 200 mM NaCl. Elution fractions containing ubiquitin were further purified by size exclusion chromatography using a S75 16/600 gel filtration column (Cytiva) with buffer B. Ubiquitin with N-terminal glycine-cysteine (GC-Ub) was purified like Ub but expressed as ubiquitin with N-terminal methionine-glycine-cysteine (MGC-Ub) with the initiator methionine being cleaved off during expression in bacteria.

### Generation of model substrates

The main experiments were performed with a substrate generated according to (*29*). The His-SUMO-Ub_4_-mEos3.2-(tail)-intein-CBD substrate was cloned into pET41, and the four linear yeast ubiquitins were replaced by human ubiquitins. The tailed variant contains a 65–amino acid–long cyclin B–derived sequence, C-terminal of mEos3.2, which is intrinsically disordered (*29*). Proteins were expressed in Rosetta 2 (DE3) and purified in a multistep procedure, starting first with a His-tag–based affinity purification. After elution with 300 mM imidazole, His-SENP2 was added (1 μg/6 μg of protein), and the mixture was dialyzed overnight to get rid of excess imidazole. Uncleaved substrate and His-SENP2 protease were removed by passage through a second HisTrap column. The flow through was then incubated with chitin resin (New England Biolabs) for 1 hour. The mixture was filtered with a gravity filter column, and the retained resin was washed with 50 ml of chitin binding buffer [20 mM Hepes (pH 8.0) and 500 mM NaCl], before charging the resin with 15 ml of chitin binding buffer supplemented with 50 mM DTT, causing intein self-cleavage overnight. The resin was eluted with 15 ml of binding buffer the next day, and the eluate was further purified by size exclusion chromatography using a S200 16/600 gel filtration column (Cytiva) with a running buffer containing buffer B and 1 mM DTT. For ubiquitylation, substrate (10 μM) was mixed with 8 μM His-mUbe1, 30 μM Ubc4-His, and 36 μM His-Ufd2 in ubiquitination buffer [50 mM Hepes (pH 7.4), 150 mM KCl, 10 mM MgCl_2_, and 10 mM ATP] at 37°C. Ubiquitin was added stepwise over the course of 4 hours up to a final concentration of 1.6 mM, and the reaction was further incubated overnight. The mixture was passed through a HisTrap column (Cytiva) to remove the His-tagged enzymes and further purified by size exclusion chromatography using a Superose 6 increase column equilibrated with 50 mM Hepes (pH 7.4), 150 mM KCl, 2 mM MgCl_2_, and 5% glycerol. Highly ubiquitinated fractions were pooled, concentrated, and stored at −80°C.

For individual experiments (analysis of yeast proteins, Ufd1 cross-links, and effect of chain length), a model substrate according to (*28*) was used. Briefly, His-Ub_2_-Eos was used for expression in Rosetta 2 (DE3) and purified by His-tag–based affinity purification followed by size exclusion chromatography using a S200 16/600 column (Cytiva). For ubiquitylation, His-Ub_2_-Eos (10 μM) was mixed with 2 μM Strep-mUbe1, 20 μM gp78-ubc7 fusion protein, and 10 mM ATP in ubiquitination buffer at 37°C. Ubiquitin was added stepwise over the course of 4 hours up to a final concentration of 400 μM, and the reaction was further incubated overnight. The ubiquitinated His-Ub_2_^n^-Eos was isolated using a HisTrap column (Cytiva) followed by size exclusion chromatography using an S200 16/600 column (Cytiva) with buffer B. Fractions were analyzed by SDS–polyacrylamide gel electrophoresis (PAGE) and separated into long, medium, and short chains

### Photoconversion of mEos3.2

Purified substrates were irradiated with a 365-nm-long-wave ultraviolet (UV) lamp (UVP Blak-Ray B-100AP) to induce a break in the sequence of mEos3.2 that causes a shift from green [excitation (Ex.), 500 nm; and emission (Em.), 520 nm] to red (Ex., 540 nm; and Em., 580 nm) fluorescence. Photoconverted Eos (red) is unable to refold after unfolding by p97, allowing it to be used as reporter for pure p97 unfolding activity. Meanwhile, intact Eos (green) rapidly refolds, leading to a steady-state level of fluorescence that is susceptible to proteasomal degradation. Samples were irradiated for 30-min on/off intervals over 4 hours at 4°C.

### Generation of fluorescently labeled free ubiquitin chains

Synthesis of K48-linked Ub chains was performed as described (*37*) in 50 mM tris (pH 8.0), 5 mM MgCl_2_, 10 mM ATP, and 0.5 mM DTT, by adding 0.5 μM E1 enzyme, 20 μM His_6_-Lipoyl-TEV-Ube2K, 0.8 mM Ub, and 0.2 mM GC-Ub. The reaction was incubated at 37°C for 4 hours and quenched by addition of 5 mM DTT and incubation for 20 min at room temperature (RT). E2 was removed by applying the reaction (after addition of 150 mM NaCl and 20 mM imidazole) over Pure Cube Ni-INDIGO beads equilibrated in 50 mM tris (pH 8.0), 150 mM NaCl, and 20 mM Imidazole. The flow through and wash were combined, acidified in 50 mM NaOAc (pH 4.5), and purified by length on a 6 ml of Resource S (Cytiva) column using a gradient up to 1 M NaCl in 50 mM NaOAc (pH 4.5) and 1 mM Tris(2-carboxyethyl)phosphine (TCEP). Peaks were neutralized using 1 ml of 1 M tris (pH 8.0) per 4 ml of pH 4.5 buffer and buffer exchanged during concentration into 20 mM Hepes (pH 7.5). For labeling, K48-Ub_5_ (containing 5 Ub and 1/5 CG-Ub) at a concentration of 100 μM Ub chain and 500 μM fluorescein-5-maleimide was incubated overnight at 4°C in 20 mM Hepes (pH 7.5). The reaction was quenched on the next morning by addition of 10 mM DTT and incubated at RT for 10 min. Fluorescein-Ub chains were acidified by dilution into 50 mM NaOAc (pH 4.5) and applied to 100 μl of SP Sepharose HP (Cytiva). After washing in 30 column volumes (CV) of 50 mM NaOAc (pH 4.5) and 20 CV of 50 mM NaOAc (pH 4.5) and 100 mM NaCl, chains were eluted by increasing the NaCl concentration to 500 mM. The buffer was exchanged to 20 mM Hepes (pH 7.5) during concentration.

### Coupled unfolding-degradation assay

Substrate unfolding by p97 coupled with degradation by the 26*S* proteasome was measured using a two-phase fluorescence assay (Ex., 500 nm; and Em., 520 nm). First, polyubiquitinated substrate (Ub_4_^n^-Eos or Ub_4_^n^-Eos-tail,100 nM), p97 (250 nM hexamer), Ufd1-Npl4 (500 nM), and accessory factor (FAF1, FAF2, UBXN7, and RAD23B; 250 nM) were mixed in degradation buffer [25 mM Hepes (pH 7.4), 100 mM KCl, 5 mM MgCl_2_, 5% glycerol, 1 mM DTT, and bovine serum albumin (BSA; 0.5 mg/ml)] at 37°C. The unfolding reaction was started by the addition of an ATPase regenerating mix [2 mM ATP, 10 mM creatine-phosphate, and creatine kinase (10 μg/ml)], and green mEos3.2 fluorescence was monitored for 40 min using a Cary-Eclipse fluorescence spectrophotometer (Varian) until a steady-state level had been reached. Proteasomal degradation was induced by the addition of 100 nM mouse 26*S* proteasome, and fluorescence was monitored for further 40 min. For the Ub_4_-Eos-Tail substrate, only direct degradation by the proteasome was measured, omitting other proteins and the unfolding phase and adding the ATP regenerating system before addition of 26*S*. Control samples were supplemented with an equal volume of UBL buffer with 40% glycerol, 1 mM DTT, and 1 mM ATP. Fluorescence curves were plotted with Excel (Microsoft) and GraphPad Prism.

### Unfolding assay

The effect of cofactors on the unfolding rate of p97 and Cdc48 without subsequent proteasomal degradation was measured using a single-phase fluorescence assay (Ex., 540 nm; and Em., 580 nm). Polyubiquitinated substrate, p97 or Cdc48 (175 nM hexamer), Ufd1-Npl4 (500 nM), and accessory factor (FAF1, FAF2, UBXN7, and Ubx5; 500 nM) were mixed in unfolding buffer [25 mM Hepes (pH 7.4), 100 mM KCl, 5 mM MgCl_2_, 5% glycerol, and 1 mM DTT] at 37°C for p97 or 30°C for Cdc48. The unfolding reaction was started by addition of ATP to 2 mM, and red mEos3.2 fluorescence was monitored for 40 min using a Cary-Eclipse fluorescence spectrophotometer (Varian).

### Labeling of Ub_4_^n^-Eos

Ub_4_^n^-Eos substrate was labeled with IRDye 800CW maleimide (LICORbio) for binding and degradation experiments tagging mostly the exposed cysteine-195 in mEos. The dye was incubated at a fivefold excess of dye/protein to 100 μM of substrate at 4°C for 1 hour. The reaction was stopped by the addition of 1 mM DTT and incubated for 10 more minutes. Excess dye molecules were removed by two rounds of buffer exchange with buffer B using Zeba desalting columns (7-kDa MWCO, Thermo Fisher Scientific).

### SDS-gel–based degradation assay

IRDye 800–labeled Ub_4_^n^-Eos (100 nM) was mixed with p97 (250 nM) and Ufd1-Npl4 (500 nM) in degradation buffer [25 mM Hepes (pH 7.4), 100 mM KCl, 5 mM MgCl2, 5% glycerol, 1 mM DTT, and BSA (0.5 mg/ml)] together with an ATPase regenerating mix [2 mM ATP, 10 mM creatine-phosphate, and creatine kinase (10 μg/ml)]. Accessory factors (FAF1, FAF2, and UBXN7; 250 nM) or bortezomib (20 μM) was added as indicated. Samples were incubated at 37°C for 1 hour, before addition of 6× SDS-sample buffer and analyzed by SDS-PAGE followed by a fluorescence scan using an odyssey fluorescence scanner (LICORbio; Ex., 785 nm; and Em., 816 to 840 nm).

### Fluorescence anisotropy

Binding of free fluorescein-labeled K48-Ub_5_ chains (500 nM) to p97-UN without or with FAF1 at indicated concentrations in unfolding assay buffer was followed in via fluorescence anisotropy. For this, fluorescence anisotropy of fluorescein was followed in a Varian Cary Eclipse spectrofluorometer (excitation wavelength, 460 nm; emission wavelength, 530 nm; excitation slit, 20 nm; emission slit, 20 nm; and temperature, 25°C). Dissociation constant (*K*_d_) values were determined by fitting to the equation for one site-specific binding (GraphPad Prism).

### Biolayer interferometry

Cysteines in the Eos moiety of poly-ubiquitylated Ub_4_-Eos were ubiquitylated using a 20-fold molar excess of EZ-Link Maleimide-PEG2-Biotin (Thermo Fisher Scientific) for 16 hours at 4°C in buffer A, and excess biotin was removed using Zeba desalting columns (7-kDa MWCO, Thermo Fisher Scientific). Biolayer interferometry assays were performed on an Octet R8 instrument (Sartorius) at 25°C in buffer A supplemented with 2% BSA and 0.25% Tween 20. After equilibration, biotinylated Ub_n_-Eos (1.25 μg/ml) was immobilized on streptavidin biosensors (60 s), baseline was recorded (120 s) before binding (60 s) of p97 or p97-Ufd1-Npl4 or p97-Ufd1-Npl4-FAF1 (312.5 to 2500 nM serial dilution), and dissociation (480 s) was recorded. Kinetics data were processed with Octet Analysis Studio 13.0.2.46. Data were aligned on the *y* axis to the last 5 s of the baseline step, and interstep correction was applied to align data to the beginning of the dissociation step. Association and dissociation steps were fitted individually to a heterogeneous ligand-binding model (two ligand-binding sites).

### Substrate-binding assay

IRDye 800–labeled Ub_4_^n^-Eos (1 μM) was mixed with different combinations of p97-His (1 μM, purified from bacteria), Ufd1-Npl4 (1 μM), and FAF1 (1 μM) in immunoprecipitation (IP) buffer [50 mM tris (pH 7.4), 150 mM KCl, 5 mM MgCl_2_, 5% glycerol, 1% Triton X-100, and 2 mM β-mercaptoethanol], together with anti-p97 (Santa Cruz Biotechnology, sc-57492; mouse monoclonal, diluted 1:1000) and GammaBind Plus Sepharose (Cytiva). After incubation with constant overhead turning at 4°C for 1 hour, the beads were washed three times with IP buffer. Bound proteins were eluted by boiling in 1.5× Laemli-Buffer at 95°C for 5 min. After SDS-PAGE, the gel was scanned with an odyssey fluorescence scanner (LICORbio; Ex., 785 nm; and Em., 816 to 840 nm) and analyzed by Western blot.

### Cross-linking of Ufd1 E143BpA

For the detection of cross-links between FAF1 and Ufd1, Ufd1-E143Bpa-Npl4 (1 μM) was mixed with p97-His (1 μM) and accessory factor (1 μM) in buffer B. Cross-links were induced by irradiation with 365 nm (CL-1000 UV cross-linker, Analytik Jena) for 30 min at 4°C. Samples were analyzed by SDS-PAGE and Western blot.

### Cell culture

HeLa Kyoto cells (RRID: CVCL_1922) were cultured in Dulbecco’s modified Eagle’s medium supplemented with 10% fetal bovine serum (FBS; PAN-Biotech) and 1% penicillin/streptomycin (PAN-Biotech). Cells were grown under standard conditions (37°C and 5% CO_2_). Cells were transfected with plasmid DNA using Lipofectamine 2000 (Thermo Fisher Scientific) according to the manufacturer’s instructions. For FAF1 transfection, the human FAF1 open reading frame was inserted into pcDNA5-FRT/TO-SH (*38*). Mutations were generated by QuikChange.

### Cell death analysis by flow cytometry

Cells were seeded on a six-well plate followed by transfection with indicated plasmids on the following day. Next day, cells were harvested by trypsinization and incubated in Hoechst 33342 (3 μg/ml) in PBS. The unstained and single-stain control for propidium iodide (PI) were incubated in PBS. Subsequently, the cells were centrifuged followed by incubation with PI (10 μg/ml) in fluorescence-activated cell sorting (FACS) buffer [1 mM EDTA, 1% FBS, and ribonuclease A (10 μg/ml) in PBS] for 15 min at RT. The unstained and single-stain control for Hoechst 33342 were incubated in FACS buffer. After staining, 10,000 events per sample were acquired at MACSQuant MQ16 flow cytometer. For cell death analysis, cells were gated for Hoechst 33342, and the percentage of PI-positive cells was determined with FlowJo Analysis software.

### AlphaFold modeling

Structural models were generated using the AlphaFold 3 web server (*39*). Structures and confidence matrices were visualized using ChimeraX 1.9 (*40*). The chain_pair_pae_min values correlate with whether two chains in a complex interact with each other and were used to determine the confidence of interactions between individual proteins within the complex.

### Statistical analysis

Data were analyzed using GraphPad Prism. Initial rates were calculated from the slope at *t* = 0 (or addition of 26*S* proteasome for degradation rates) as determined by one-phase nonlinear regression over the subsequent 5 to 10 min. In cases where the change in fluorescence showed no curvature, linear regression was used instead to determine initial rates. Experiments were performed with *n* = 2 or *n* = 3 independent experiments as indicated in the figure legends, and error bars denote SD unless stated otherwise. Statistical significance was determined using one-way analysis of variance (ANOVA) or unpaired two-tailed Student’s *t* test, as indicated in the figure legends.

## Supplementary Materials

### This PDF file includes:

Figs. S1 to S4 Tables S1 and S2 References
