# Supplementary material for: The accessory adapters FAF1, FAF2, and UBXN7 accelerate proteasomal degradation by increasing prior p97-mediated substrate unfolding
Source: Sci Adv. 2026 Mar 6;12(10):eaea7381. doi: 10.1126/sciadv.aea7381 (PMC12965308; doi:10.1126/sciadv.aea7381)
Supplement: Supplementary file 1 — Figs. S1 to S4 Tables S1 and S2 References [file sciadv.aea7381_sm.pdf]

Science Advances
AAAS

Supplementary Materials for

The accessory adapters FAF1, FAF2, and UBXN7 accelerate proteasomal degradation by increasing prior p97-mediated substrate unfolding

Matthias Kracht *et al.*

Corresponding author: Hemmo Meyer, hemmo.meyer@uni-due.de

Sci. Adv. **12**, eaea7381 (2026)
DOI: 10.1126/sciadv.aea7381

This PDF file includes:

Figs. S1 to S4
Tables S1 and S2
References

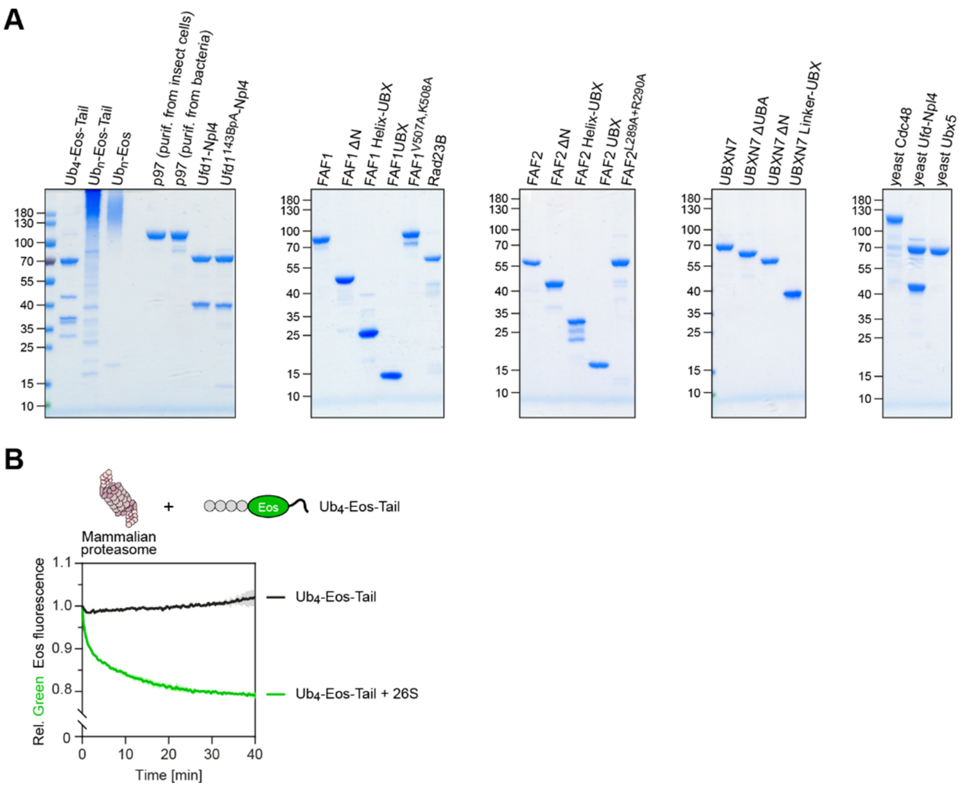

Fig. S1. Quality control of proteins used in the study.

(**A**) Coomassie gels of purified proteins.

(**B**) Time course of fluorescence intensities of ubiquitylated Ub4-Eos with a short disordered C-terminal peptide tail incubated without or with the proteasome. Note that only the substrate with proteasome is degraded, as indicated by the loss of green fluorescence. n = 2; mean ± SD.

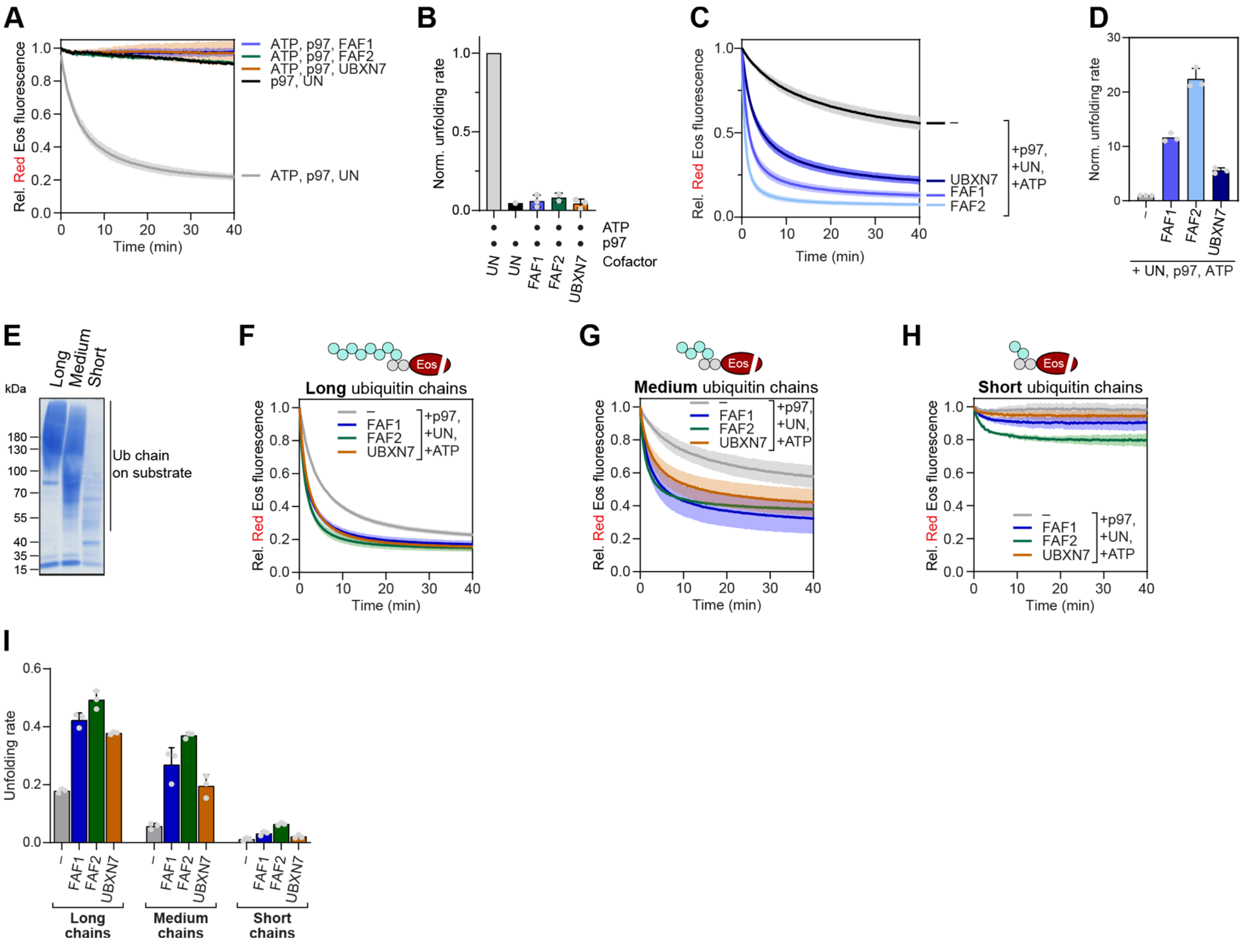

Fig. S2. Experiments related to Fig. 2.

(**A**) Ufd1-Npl4 (UN) is essential for stimulation by accessory adapters. Unfolding was monitored with protein combinations as indicated in the presence or absence of ATP. Note that p97 with accessory adapters alone without Ufd1-Npl4 do not support unfolding. n = 3 (for FAF1, UBXN7), n = 2 (for FAF2, UN+ATP), n = 1 (UN).

(**B**) Quantification of (A).

(**C and D**) Unfolding reactions without (-) or with indicated accessory adapters at high concentrations of p97 (1 µM), UN (2 µM), accessory adapters (2 µM), and substrate (600 nM). Quantification, n = 3, error bars represent S.D.

(**E**) Coomassie-stained SDS-gel showing substrate fractions with different ubiquitin chain lengths.

(**F to H**) Unfolding reactions with substrates harboring different ubiquitin chain lengths shown in (E) in the presence of p97 and Ufd1-Npl4 alone supplemented with indicated accessory adapters. Note that accessory adapters stimulate unfolding of substrates with short and long ubiquitin chains. n = 3.

(**G**) Quantification of (F to H). n = 3.

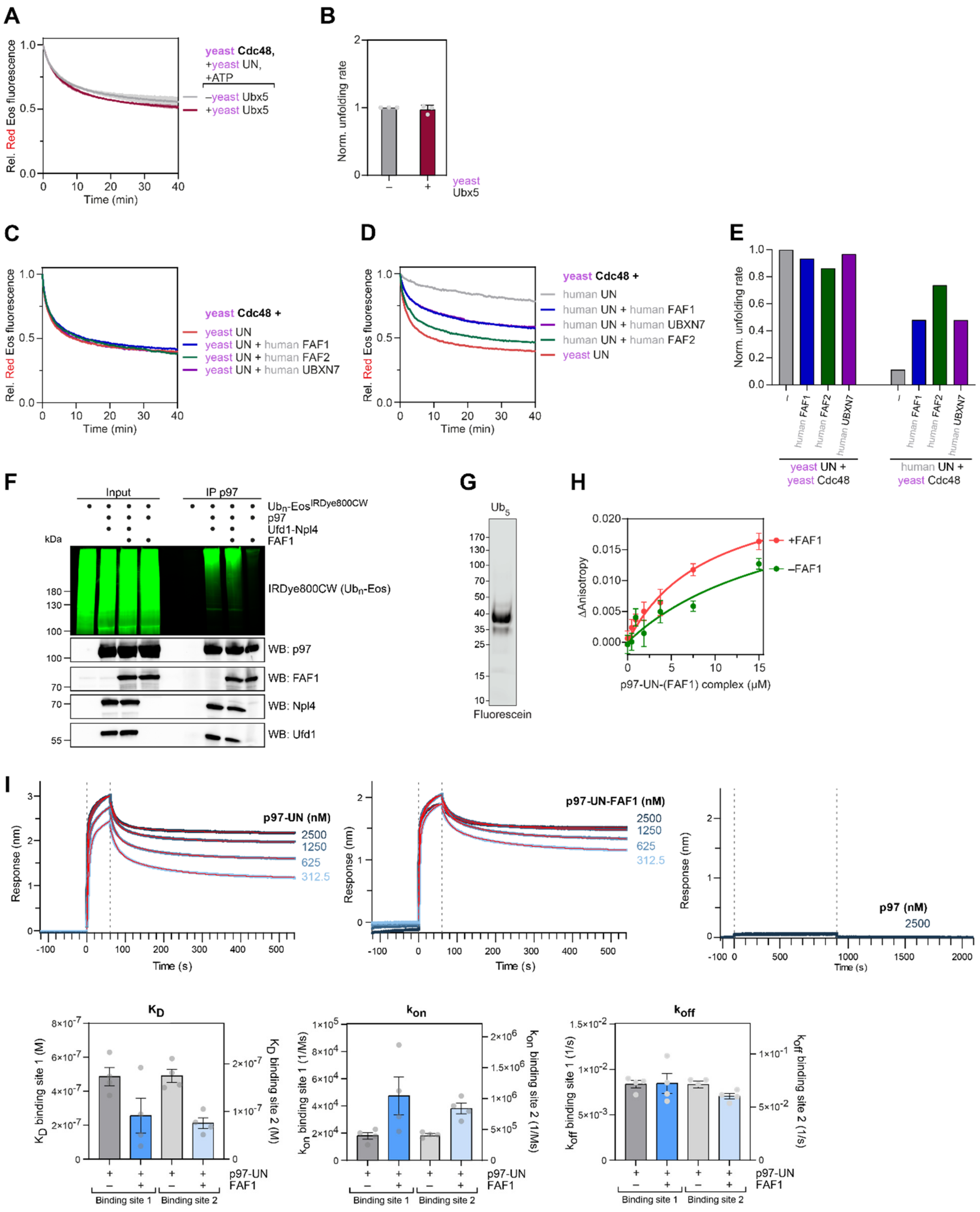

A
Rel. Red Eos fluorescence
Time (min)
yeast Cdc48,
+yeast UN,
+ATP
–yeast Ubx5
+yeast Ubx5
B
Norm. unfolding rate
yeast
Ubx5
C
Rel. Red Eos fluorescence
Time (min)
yeast Cdc48 +
yeast UN
yeast UN + human FAF1
yeast UN + human FAF2
yeast UN + human UBXN7
D
Rel. Red Eos fluorescence
Time (min)
yeast Cdc48 +
human UN
human UN + human FAF1
human UN + human UBXN7
human UN + human FAF2
yeast UN
E
Norm. unfolding rate
human FAF1
human FAF2
human UBXN7
yeast UN +
yeast Cdc48
human UN +
yeast Cdc48
F
Input
IP p97
Ubn-EosIRDye800CW
p97
Ufd1-Npl4
FAF1
kDa
IRDye800CW (Ubn-Eos)
WB: p97
WB: FAF1
WB: Npl4
WB: Ufd1
G
Ub5
Fluorescein
H
ΔAnisotropy
+FAF1
–FAF1
p97-UN-(FAF1) complex (μM)
I
Response (nm)
Time (s)
p97-UN (nM)
2500
1250
625
312.5
p97-UN-FAF1 (nM)
2500
1250
625
312.5
p97 (nM)
2500
KD
KD binding site 1 (M)
KD binding site 2 (M)
kon
kon binding site 1 (1/Ms)
kon binding site 2 (1/Ms)
koff
koff binding site 1 (1/s)
koff binding site 2 (1/s)
p97-UN
FAF1
Binding site 1
Binding site 2

Fig. S3: Analysis of substrate binding and insertion, as well as yeast protein activities.

(**A**) Unfolding activity of yeast Cdc48-Ufd1-Npl4 is not stimulated by the yeast accessory adapter Ubx5. Unfolding assays monitoring red Eos fluorescence as indicated. n = 3.

(**B**) Quantification of (A). n = 3.

(**C**) Substrate unfolding by yeast Cdc48 and yeast Ufd1-Npl4 is not affected by human accessory adapters. Unfolding assays as indicated monitoring red Eos fluorescence. n = 1.

(**D**) Unfolding by yeast Cdc48 with human Ufd1-Npl4 is slow but is stimulated by human accessory adapters. Unfolding assays as indicated monitoring red Eos fluorescence. The curve for yeast Ufd1-Npl4 was added from (C) for comparison. n = 1.

(**E**) Quantification of (C and D). Normalization to the activity of yeast Cdc48 and yeast Ufd1-Npl4. n = 1.

(**F**) Binding assays of ubiquitylated substrates with p97 complexes. The fluorescently labelled substrate was incubated with indicated combinations of proteins. p97 was immuno-isolated and associated proteins analyzed in an SDS-gel by fluorescence scan and Western blot. Note that FAF1 does not stimulate substrate binding to p97.

(**G**) Unanchored lysine-48-linked ubiquitin chains were generated enzymatically and labelled with fluorescein.

(**H**) Fluorescence polarization of the ubiquitin chains in (G) was determined after binding to p97-Ufd1-Npl4 in the presence or absence of FAF1 as indicated. K_D values without FAF1 (19.4 ± 3.8 μM) and with FAF1 (9.3 ± 2.1 μM) were determined by nonlinear regression. Error bars represent SEM from 5 technical replicates.

(**I**) Bio-layer interferometry sensorgrams for p97 complex binding to ubiquitylated substrate. Biotinylated Ub_n-Eos was immobilized and protein binding (first dashed line) and dissociation (second dashed line) was followed for indicated concentrations of p97 or p97-Ufd1-Npl4 (UN) without or with FAF1 as indicated. Red lines indicate fits for heterogenous ligand binding assuming two binding sites. Fitted parameters for K_D, k_{on} and k_{off} (means ± SEM; n = 4 concentrations) were plotted for the two binding sites.

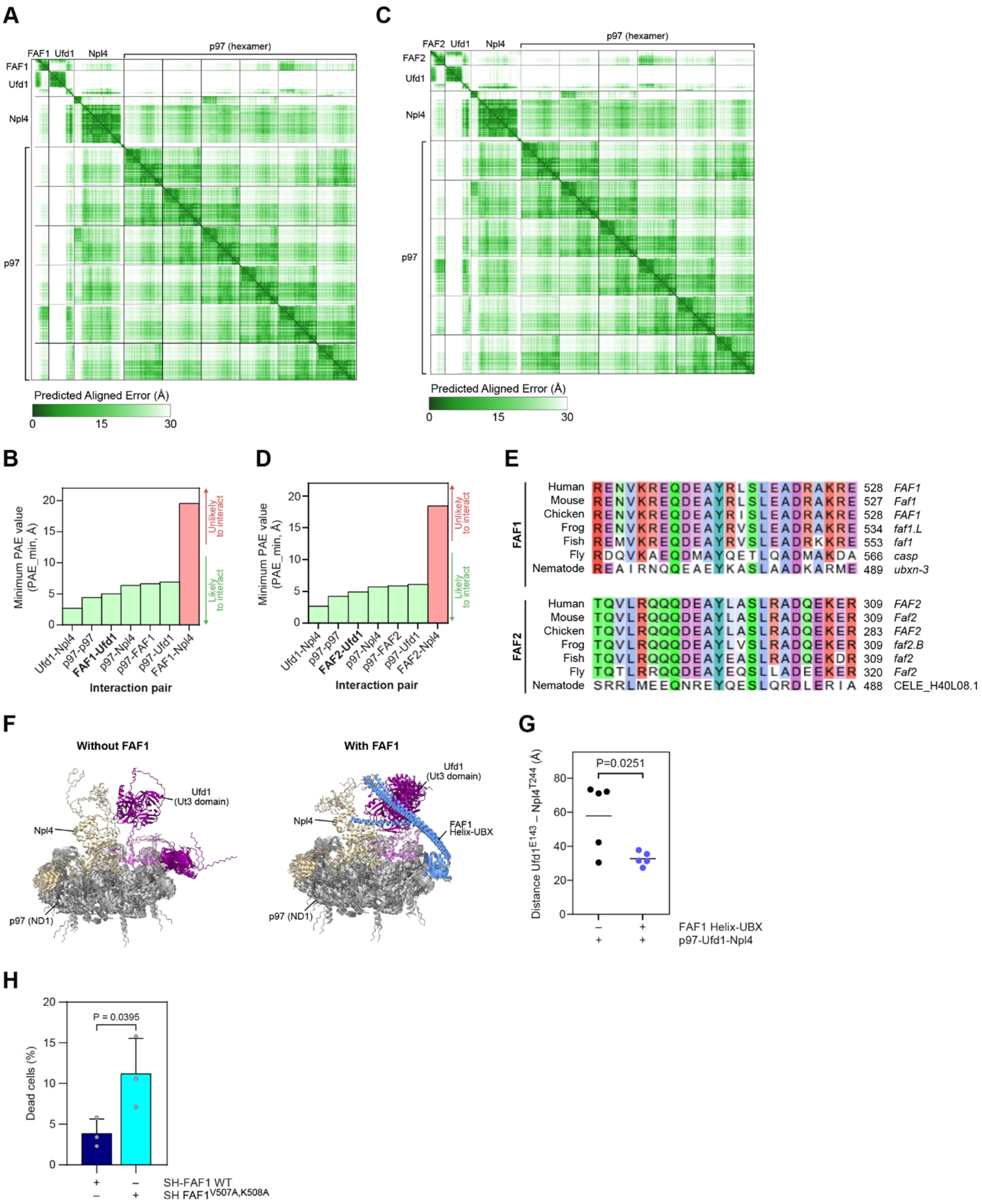
A
FAF1
Ufd1
Npl4
p97 (hexamer)
p97
Predicted Aligned Error (Å)
0
15
30
C
FAF2
Ufd1
Npl4
p97 (hexamer)
p97
Predicted Aligned Error (Å)
0
15
30
B
Minimum PAE value (PAE_min, Å)
Unlikely to interact
Likely to interact
Ufd1-Npl4
p97-p97
FAF1-Ufd1
p97-Npl4
p97-FAF1
p97-Ufd1
FAF1-Npl4
Interaction pair
D
Minimum PAE value (PAE_min, Å)
Unlikely to interact
Likely to interact
Ufd1-Npl4
p97-p97
FAF2-Ufd1
p97-Npl4
p97-FAF2
p97-Ufd1
FAF2-Npl4
Interaction pair
E
FAF1
Human RENVKREQDEAYRLSLEADRAKRE 528 FAF1
Mouse RENVKREQDEAYRLSLEADRAKRE 527 Faf1
Chicken RENVKREQDEAYRISLEADRAKRE 528 FAF1
Frog RENVKREQDEAYRVSLEADRAKRE 534 faf1.L
Fish REMVKREQDEAYRVSLEADRKKRE 553 faf1
Fly RDQVKAEQDMAYQETLQADMAKDA 566 casp
Nematode REAIRNQQEAEYKASLAADKARME 489 ubxn-3
FAF2
Human TQVLRQQQDEAYLASLRADQEKER 309 FAF2
Mouse TQVLRQQQDEAYLASLRADQEKER 309 Faf2
Chicken TQVLRQQQDEAYLASLRADQEKER 283 FAF2
Frog TQVLRQQQDEAYLVSLRADQEKER 309 faf2.B
Fish TQVLRQQQDEAYEASLRADQEKDR 309 faf2
Fly TQTLRRQQDEAYEQSLLADEEKER 320 Faf2
Nematode SRRLMEEQNREYQESLQRDLERIA 488 CELE_H40L08.1
F
Without FAF1
With FAF1
Ufd1 (Ut3 domain)
Npl4
p97 (ND1)
FAF1 Helix-UBX
G
P=0.0251
Distance Ufd1E143 – Npl4T244 (Å)
FAF1 Helix-UBX
p97-Ufd1-Npl4
H
P = 0.0395
Dead cells (%)
SH-FAF1 WT
SH FAF1V507A,K508A

Fig. S4. Data related to Fig. 3A and B.

(**A**) Predicted aligned error (PAE) plots for the AlphaFold 3 model of the complex comprising a hexamer of p97 (aa 1-469), Ufd1, Npl4 and FAF1.

(**B**) Plot of minimum PAE values for each protein-protein interaction in the AlphaFold 3-modeled complex with FAF1. Smaller values indicate higher confidence of the modeled interface.

(**C**) Predicted aligned error (PAE) plots for the AlphaFold 3 model of the complex comprising a hexamer of p97 (aa 1-469), Ufd1, Npl4 and FAF2.

(**D**) Plot of minimum PAE values for each protein-protein interaction in the AlphaFold 3-modeled complex with FAF2. Smaller values indicate higher confidence of the modeled interface.

(**E**) Multiple sequence alignments of the Ufd1 interface in FAF1 and FAF2. Protein sequences of FAF1 or FAF2 homologs were identified in *Mus musculus* (mouse), *Gallus gallus* (chicken), *Xenopus laevis* (frog), *Danio rerio* (fish), *Drosophila melanogaster* (fly) and *Caenorhabditis elegans* (nematode) and aligned with Clustal Omega. Gene names and residue numbers within the respective sequences are indicated. Residues are colored according to clustal2 color scheme.

(**F**) Overlay of five AlphaFold 3 models of hexameric p97 (aa 1-469), Ufd1-Npl4 without and with FAF1 Helix-UBX. Models were aligned to the Ufd1-Npl4 interface (residues 267-269 in Ufd1). Note that the Ut3 domain is consistently predicted diagonally above the p97 pore in the presence but not in the absence of FAF1.

(**G**) Distance between Ufd1 and Npl4 in the AlphaFold 3 models displayed in (F). The Cα distance between the FAF1 binding site of Ufd1 (E143 in the Ut3 domain of Ufd1) and the binding site of the ubiquitin branch point in Npl4 (T244 in Npl4) was measured. Significance was calculated with an unpaired t test. n = 5 models. Lines indicate means.

(**H**) Overexpression of the FAF1 (V507A, K508A) mutant in HeLa cells induces increased cell death. Indicated FAF1 variants were transiently transfected and cell death was determined by influx of propidium iodide and measured by flow cytometry. Values normalized to cells transfected with empty vector control. n = 3 biological replica. Significance was tested by two-tailed paired t test. Error bars represent S.D.

Table S1. Plasmids used in this study.

Name	Source
pFL His-p97	van den Boom *et al*, 2016 (*36)*
pET41 p97-His	This study
pET41 Ufd1-His	van den Boom *et al*, 2016 (*36*)
pET41 Ufd1-His E143TAG	This study
pET15 p97-SBP-His E314Amb	Weith *et al*, 2018 (41)
pET41 Npl4	van den Boom *et al*, 2016 (*36*)
pET23 Ubiquitin	Meyer *et al,* 2002 (*9*)
pET28 His-FAF1	This study
pET28 His-FAF1 ΔN (Δ1-270)	This study
pET28 His-FAF1 Helix-UBX (481-650)	This study
pET28 His-FAF1 UBX (569-650)	This study
pET28 His-FAF1 V507A K508A	This study
pET28 His-FAF2 ΔTMD	This study
pET28 His-FAF2 ΔTMD ΔN (Δ1-131)	This study
pET28 His-FAF2 ΔTMD Helix-UBX (276-445)	This study
pET28 His-FAF2 ΔTMD UBX (350-445)	This study
pET28 His-FAF2 ΔTMD L289A R290A	This study
pET28 His-UBXN7	This study
pET28 His-UBXN7 ΔUBA (Δ2-54)	This study
pET28 His-UBXN7 ΔN (Δ1-100)	This study
pET28 His-UBXN7 Linker-UBX (Δ1-265)	This study
pET41 His-SUMO-Ub_4-mEos3.2-Inteinein-CBD	Adapted from Olszewski *et al*, 2019 (*29*)
pET41 His-SUMO-Ub_4-mEos3.2-Tail-Int-CBD	Adapted from Olszewski *et al*, 2019 (*29*)
pET28 His-Ub_2-mEos3.2	Adapted from Blythe *et al,* 2017 (*28*)
pGEX6P1 GST-Rad23B	This study
pET41 His-Cdc48	This study
pET41 Ufd1-His (*S. cerevisiae*)	This study
pET41 Npl4 (*S. cerevisiae*)	This study
pET28 His-Ubx5 (S. *cerevisiae*)	This study
pcDNA5-FRT/TO-SH	Hülsmann *et al*, 2018 (*38*)
pcDNA5-FRT/TO-SH-FAF1 WT	This study
pcDNA5-FRT/TO-SH-FAF1 V507A,K508A	This study

Table S2. Antibodies used in this study.

Primary antibody	Source	Secondary antibody
anti-FAF1	van den Boom *et al,* 2016 (*35*)	anti-rabbit-HRP
anti-FAF2	Proteintech, 16251-1-AP	anti-rabbit-HRP
anti-Ufd1	BD Bioscience, 611642	anti-mouse-HRP
anti-Npl4	Atlas Antibodies HPA021560	anti-rabbit-HRP
anti-p97	Santa Cruz, sc-57492	anti-mouse-HRP

REFERENCES

1. Y. Ye, W. K. Tang, T. Zhang, D. Xia, A. Mighty, "Protein extractor" of the cell: Structure and function of the p97/CDC48 ATPase. *Front. Mol. Biosci.* **4**, 39 (2017).

2. A. Stolz, W. Hilt, A. Buchberger, D. H. Wolf, Cdc48: A power machine in protein degradation. *Trends Biochem. Sci.* **36**, 515–523 (2011).

3. H. Meyer, J. van den Boom, Targeting of client proteins to the VCP/p97/Cdc48 unfolding machine. *Front. Mol. Biosci.* **10**, 1142989 (2023).

4. E. C. Twomey, Z. Ji, T. E. Wales, N. O. Bodnar, S. B. Ficarro, J. A. Marto, J. R. Engen, T. A. Rapoport, Substrate processing by the Cdc48 ATPase complex is initiated by ubiquitin unfolding. *Science* **365**, eaax1033 (2019).

5. I. Cooney, H. Han, M. G. Stewart, R. H. Carson, D. T. Hansen, J. H. Iwasa, J. C. Price, C. P. Hill, P. S. Shen, Structure of the Cdc48 segregase in the act of unfolding an authentic substrate. *Science* **365**, 502–505 (2019).

6. M. Pan, Y. Yu, H. Ai, Q. Zheng, Y. Xie, L. Liu, M. Zhao, Mechanistic insight into substrate processing and allosteric inhibition of human p97. *Nat. Struct. Mol. Biol.* **28**, 614–625 (2021).

7. J. van den Boom, G. Marini, H. Meyer, H. R. Saibil, Structural basis of ubiquitin-independent PP1 complex disassembly by p97. *EMBO J.* **42**, e113110 (2023).

8. I. Cooney, H. L. Schubert, K. Cedeno, O. N. Fisher, R. Carson, J. C. Price, C. P. Hill, P. S. Shen, Visualization of the Cdc48 AAA+ ATPase protein unfolding pathway. *Nat. Commun.* **15**, 7505 (2024).

9. H. H. Meyer, Y. Wang, G. Warren, Direct binding of ubiquitin conjugates by the mammalian p97 adaptor complexes, p47 and Ufd1-Npl4. *EMBO J.* **21**, 5645–5652 (2002).

10. Y. Ye, H. H. Meyer, T. A. Rapoport, Function of the p97-Ufd1-Npl4 complex in retrotranslocation from the ER to the cytosol: Dual recognition of nonubiquitinated polypeptide segments and polyubiquitin chains. *J. Cell Biol.* **162**, 71–84 (2003).

11. N. O. Bodnar, T. A. Rapoport, Molecular mechanism of substrate processing by the Cdc48 ATPase complex. *Cell* **169**, 722–735.e9 (2017).

12. C. Williams, K. C. Dong, C. Arkinson, A. Martin, The Ufd1 cofactor determines the linkage specificity of polyubiquitin chain engagement by the AAA+ ATPase Cdc48. *Mol. Cell* **83**, 759–769.e7 (2023).

13. A. Beskow, K. B. Grimberg, L. C. Bott, F. A. Salomons, N. P. Dantuma, P. Young, A conserved unfoldase activity for the p97 AAA-ATPase in proteasomal degradation. *J. Mol. Biol.* **394**, 732–746 (2009).

14. S. Prakash, L. Tian, K. S. Ratliff, R. E. Lehotzky, A. Matouschek, An unstructured initiation site is required for efficient proteasome-mediated degradation. *Nat. Struct. Mol. Biol.* **11**, 830–837 (2004).

15. A. Buchberger, H. Schindelin, P. Hanzelmann, Control of p97 function by cofactor binding. *FEBS Lett.* **589**, 2578–2589 (2015).

16. L. Stach, P. S. Freemont, The AAA+ ATPase p97, a cellular multitool. *Biochem. J.* **474**, 2953–2976 (2017).

17. O. Neuber, E. Jarosch, C. Volkwein, J. Walter, T. Sommer, Ubx2 links the Cdc48 complex to ER-associated protein degradation. *Nat. Cell Biol.* **7**, 993–998 (2005).

18. C. Schuberth, A. Buchberger, Membrane-bound Ubx2 recruits Cdc48 to ubiquitin ligases and their substrates to ensure efficient ER-associated protein degradation. *Nat. Cell Biol.* **7**, 999–1006 (2005).

19. J. Zheng, Y. Cao, J. Yang, H. Jiang, UBXD8 mediates mitochondria-associated degradation to restrain apoptosis and mitophagy. *EMBO Rep.* **23**, e54859 (2022).

20. G. Alexandru, J. Graumann, G. T. Smith, N. J. Kolawa, R. Fang, R. J. Deshaies, UBXD7 binds multiple ubiquitin ligases and implicates p97 in HIF1α turnover. *Cell* **134**, 804–816 (2008).

21. A. Franz, P. A. Pirson, D. Pilger, S. Halder, D. Achuthankutty, H. Kashkar, K. Ramadan, T. Hoppe, Chromatin-associated degradation is defined by UBXN-3/FAF1 to safeguard DNA replication fork progression. *Nat. Commun.* **7**, 10612 (2016).

22. R. Sonneville, S. P. Moreno, A. Knebel, C. Johnson, C. J. Hastie, A. Gartner, A. Gambus, K. Labib, CUL-2(LRR-1) and UBXN-3 drive replisome disassembly during DNA replication termination and mitosis. *Nat. Cell Biol.* **19**, 468–479 (2017).

23. Y. Xia, R. Fujisawa, T. D. Deegan, R. Sonneville, K. P. M. Labib, TIMELESS-TIPIN and UBXN-3 promote replisome disassembly during DNA replication termination in Caenorhabditis elegans. *EMBO J.* **40**, e108053 (2021).

24. O. V. Kochenova, S. Mukkavalli, M. Raman, J. C. Walter, Cooperative assembly of p97 complexes involved in replication termination. *Nat. Commun.* **13**, 6591 (2022).

25. R. Fujisawa, C. Polo Rivera, K. P. M. Labib, Multiple UBX proteins reduce the ubiquitin threshold of the mammalian p97-UFD1-NPL4 unfoldase. *eLife* **11**, e76763 (2022).

26. H. Li, Z. Ji, J. A. Paulo, S. P. Gygi, T. A. Rapoport, Bidirectional substrate shuttling between the 26S proteasome and the Cdc48 ATPase promotes protein degradation. *Mol. Cell* **84**, 1290–1303.e7 (2024).

27. H. C. Besche, W. Haas, S. P. Gygi, A. L. Goldberg, Isolation of mammalian 26S proteasomes and p97/VCP complexes using the ubiquitin-like domain from HHR23B reveals novel proteasome-associated proteins. *Biochemistry* **48**, 2538–2549 (2009).

28. E. E. Blythe, K. C. Olson, V. Chau, R. J. Deshaies, Ubiquitin- and ATP-dependent unfoldase activity of P97/VCP*NPLOC4*UFD1L is enhanced by a mutation that causes multisystem proteinopathy. *Proc. Natl. Acad. Sci. U.S.A.* **114**, E4380–E4388 (2017).

29. M. M. Olszewski, C. Williams, K. C. Dong, A. Martin, The Cdc48 unfoldase prepares well-folded protein substrates for degradation by the 26S proteasome. *Commun. Biol.* **2**, 29 (2019).

30. H. H. Meyer, J. G. Shorter, J. Seemann, D. Pappin, G. Warren, A complex of mammalian Ufd1 and Npl4 links the AAA-ATPase, p97, to ubiquitin and nuclear transport pathways. *EMBO J.* **19**, 2181–2192 (2000).

31. S. Park, R. Isaacson, H. T. Kim, P. A. Silver, G. Wagner, Ufd1 exhibits the AAA-ATPase fold with two distinct ubiquitin interaction sites. *Structure* **13**, 995–1005 (2005).

32. R. M. Bruderer, C. Brasseur, H. H. Meyer, The AAA ATPase p97/VCP interacts with its alternative co-factors, Ufd1-Npl4 and p47, through a common bipartite binding mechanism. *J. Biol. Chem.* **279**, 49609–49616 (2004).

33. M. Pan, Q. Zheng, Y. Yu, H. Ai, Y. Xie, X. Zeng, C. Wang, L. Liu, M. Zhao, Seesaw conformations of Npl4 in the human p97 complex and the inhibitory mechanism of a disulfiram derivative. *Nat. Commun.* **12**, 121 (2021).

34. X. Y. Huo, D. Liu, R. Zou, Z. P. Li, Y. Li, L. Pan, Y. Zhang, Z. R. Zhang, A UBH-UBX module amplifies p97/VCP's unfolding power to facilitate protein extraction and degradation. *Nat. Commun.* **16**, 10162 (2025).

35. J. W. Chin, A. B. Martin, D. S. King, L. Wang, P. G. Schultz, Addition of a photocrosslinking amino acid to the genetic code of Escherichiacoli. *Proc. Natl. Acad. Sci. U.S.A.* **99**, 11020–11024 (2002).

36. J. van den Boom, M. Wolf, L. Weimann, N. Schulze, F. Li, F. Kaschani, A. Riemer, C. Zierhut, M. Kaiser, G. Iliakis, H. Funabiki, H. Meyer, VCP/p97 extracts sterically trapped Ku70/80 rings from DNA in double-strand break repair. *Mol. Cell* **64**, 189–198 (2016).

37. L. Kiss, L. C. James, B. A. Schulman, UbiREAD deciphers proteasomal degradation code of homotypic and branched K48 and K63 ubiquitin chains. *Mol. Cell* **85**, 1467–1476.e6 (2025).

38. J. Hulsmann, B. Kravic, M. Weith, M. Gstaiger, R. Aebersold, B. C. Collins, H. Meyer, AP-SWATH reveals direct involvement of VCP/p97 in integrated stress response signaling through facilitating CReP/PPP1R15B degradation. *Mol. Cell. Proteomics* **17**, 1295–1307 (2018).

39. J. Abramson, J. Adler, J. Dunger, R. Evans, T. Green, A. Pritzel, O. Ronneberger, L. Willmore, A. J. Ballard, J. Bambrick, S. W. Bodenstein, D. A. Evans, C. C. Hung, M. O'Neill, D. Reiman, K. Tunyasuvunakool, Z. Wu, A. Zemgulyte, E. Arvaniti, C. Beattie, O. Bertolli, A. Bridgland, A. Cherepanov, M. Congreve, A. I. Cowen-Rivers, A. Cowie, M. Figurnov, F. B. Fuchs, H. Gladman, R. Jain, Y. A. Khan, C. M. R. Low, K. Perlin, A. Potapenko, P. Savy, S. Singh, A. Stecula, A. Thillaisundaram, C. Tong, S. Yakneen, E. D. Zhong, M. Zielinski, A. Zidek, V. Bapst, P. Kohli, M. Jaderberg, D. Hassabis, J. M. Jumper, Accurate structure prediction of biomolecular interactions with AlphaFold 3. *Nature* **630**, 493–500 (2024).

40. E. C. Meng, T. D. Goddard, E. F. Pettersen, G. S. Couch, Z. J. Pearson, J. H. Morris, T. E. Ferrin, UCSF ChimeraX: Tools for structure building and analysis. *Protein Sci.* **32**, e4792 (2023).

41. M. Weith, J. Seiler, J. van den Boom, M. Kracht, J. Hulsmann, I. Primorac, J. Del Pino Garcia, F. Kaschani, M. Kaiser, A. Musacchio, M. Bollen, H. Meyer, Ubiquitin-independent disassembly by a p97 AAA-ATPase complex drives PP1 holoenzyme formation. *Mol. Cell* **72**, 766–777.e6 (2018).
